# Supplementary material for: The causal relationship between the human gut microbiota and pyogenic arthritis: a Mendelian randomization study
Source: Front Cell Infect Microbiol. 2024 Nov 26;14:1452480. doi: 10.3389/fcimb.2024.1452480 (PMC11629706; doi:10.3389/fcimb.2024.1452480)
Supplement: Supplementary file 1 [file DataSheet1.zip › mendelian test/ebi-a-GCST90027710.csv_forest.pdf]

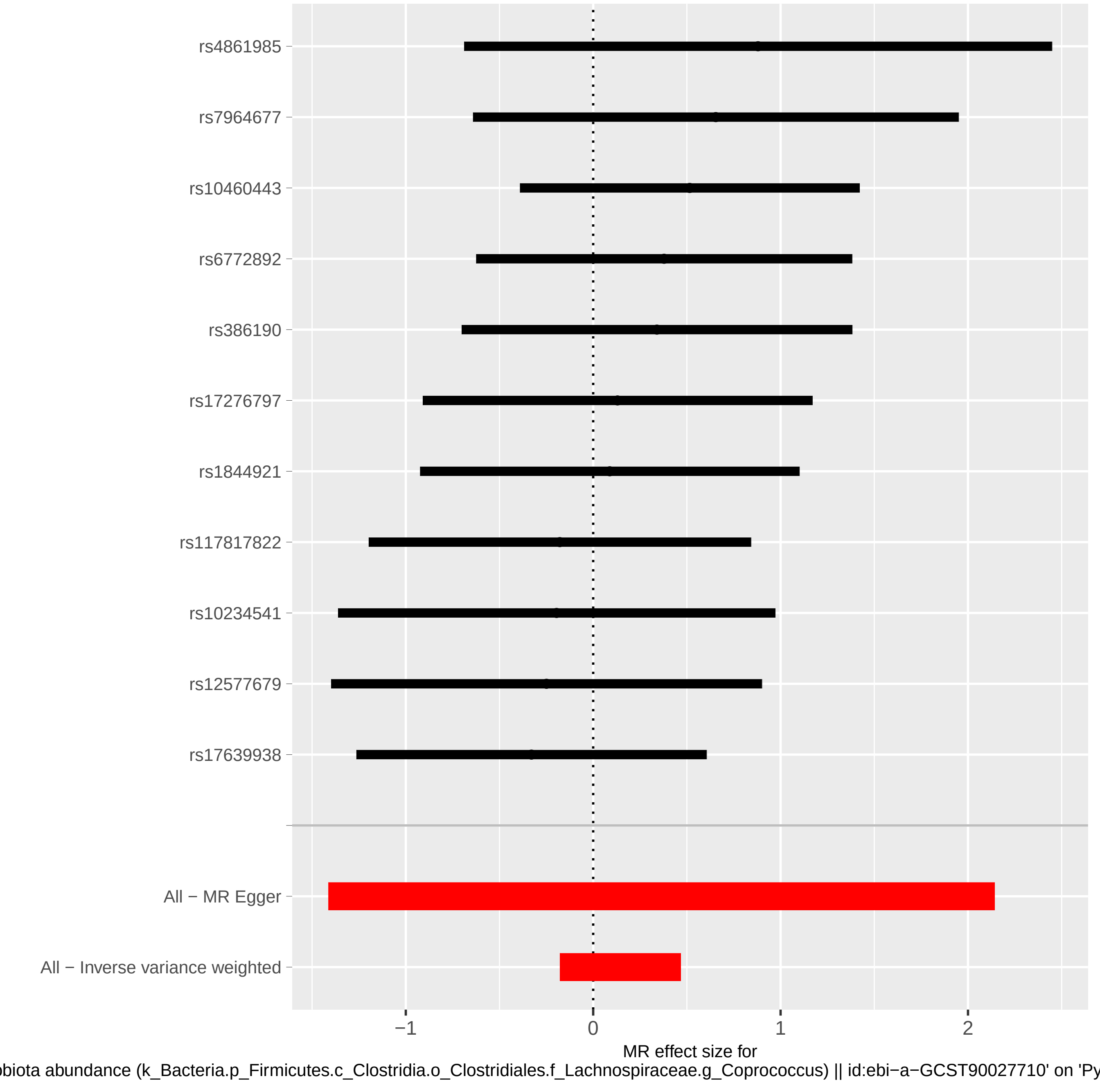
rs4861985
rs7964677
rs10460443
rs6772892
rs386190
rs17276797
rs1844921
rs117817822
rs10234541
rs12577679
rs17639938
All − MR Egger
All − Inverse variance weighted
−1
0
1
2
MR effect size for
biota abundance (k_Bacteria.p_Firmicutes.c_Clostridia.o_Clostridiales.f_Lachnospiraceae.g_Coprococcus) || id:ebi−a−GCST90027710' on 'Py
